# A case study: a continuous improvement project of lecturing skills for clinical teachers in Chinese residency standardized training

**DOI:** 10.1186/s12909-022-03311-z

**Published:** 2022-04-11

**Authors:** Xiaoliang Sun, Min Ding, Xi Luo, Baoli Kang, Yaqin Zhu, Zengguang Xu, Chi Chen

**Affiliations:** 1 Teaching and Training Department, Shanghai East Hospital, Tongji University School of Medicine, No.1800 Yuntai Road, Pudong New Area, Shanghai, 200123 China; 2 Shanghai East Hospital, Tongji University School of Medicine, No.1800 Yuntai Road, Pudong New Area, Shanghai, 200123 China

**Keywords:** Clinical teacher for the residency standardized training, Lecturing skills improvement, ADDIE, Kirkpatrick’s evaluation, Training transference

## Abstract

**Background:**

Aiming at the poor quality of small lectures due to the lack of lecturing skills of the clinical teachers in residency standardized training, the Teaching and Training Department of Shanghai East Hospital set up a continuous improvement project of lecturing skills for the clinical teachers to search for effective ways to improve lecture quality, then the effect was evaluated.

**Methods:**

Based on the ADDIE model of training design, the department conducted the project in accordance with a process of analysis, design, development, implementation and evaluation. A special course “Clinical Teacher Presentation Training” (CTPT) was developed to convey and train the 5 key behaviors in presentation to improving lecture quality of the clinical teachers. Ninety-nine clinical teachers who give lectures to the residents were recruited as subjects for the project. Adopted the model of “intensive training + practice transference” to strengthen lecturing skills, and applied the Kirkpatrick Four Levels to evaluate the effect of the project from multi-role and multi-stage.

**Results:**

The training satisfaction of the CTPT course from the subjects reaches 100%. The subjects have a high degree of knowledge acquisition through CTPT and the knowledge of the 5 key behaviors has been actually used in their lectures at the stage of practice transference. Comparing the data before training and after transference, it is found that the average increasing of the subjects’ 5 key behavior scores made by teaching secretaries is 14.12 points (14.12%) and that of the subjects’ self-efficacy scores is 9.31 points (9.31%); the performance values were modeling based on the scores from different types of evaluators and increased by an average of 12.61 points (12.61%); and the star ratings of the overall performance increased by an average of 1.17 points (23.4%). The results showed statistically difference (*P* < 0.001).

**Conclusions:**

The project effectively promoted the improvement of the clinical teachers’ lecturing skills and the quality of small lectures.

**Supplementary Information:**

The online version contains supplementary material available at 10.1186/s12909-022-03311-z.

## Background

In 2014, the Chinese residency standardized training was officially launched nationwide [1]. As the main body of education, the teaching ability of clinical teachers directly affect the level and the quality of residents’ training in hospital. The Chinese Medical Doctor Association (CMDA) has specific regulations on the teaching activities of residency standardized training. As one of the three regular teaching activities, the clinical “small lecture” is an important approach to acquire medical knowledge for residents in Chinese residency standardized training, with a problem-based learning (PBL) combined lecture-based learning (LBL) teaching model [2–4]. Clinical teachers provide supplementary materials to the residents through this 1 h oral lecture, usually including the differential diagnosis of specialty symptoms and signs, the latest developments and guidelines of the disease, etc. Therefore, the teaching ability of the clinical teachers will directly affect the quality of small lectures.

As a residency standardized training base accredited by National Health Commission of the People’s Republic of China, Shanghai East Hospital has been committed to training faculty’s teaching capabilities. In March 2019, the Teaching and Training Department of Shanghai East Hospital conducted a survey on the quality of teaching activities and the teaching ability of clinical teachers. The results are as follows:

1. Residents vote: 300 residents in training voted on “The Most Satisfying Teaching Activity”, and the results show that the least satisfaction is the “small lecture”, only 8% (24/300) (Table 1).

**Table 1:** Voting for the most satisfactory quality of teaching activities (*n* = 300)

Teaching activity	*n*(%)
Simulation course	116 (38.67)
Bedside teaching	85 (28.33)
Case discussion	40 (13.33)
Teaching rounds	35 (11.67)
Small lecture	24 (8.00)

2. Clinical teacher survey: 66.03% (103/156) of the clinical teachers chose “lecture skills” in the survey of “The Teaching Skills That You Need to Improve Most” (Table 2).

**Table 2:** Teaching skills that clinical teachers need to improve most (*n* = 156)

Teaching skills	*n*(%)
Lecturing skills	103 (66.03)
Assessment skills	34 (21.79)
Management skills	16 (10.26)
Other	3 (1.92)

The survey results show that the quality of small lectures is not satisfactory, and the lecturing skills of clinical teachers need to be improved urgently. The Teaching and Training Department further analyzed the problems, the following two reasons were discovered:

- Clinical teachers lack theoretical knowledge and skills of good presentation;
- Due to the incomplete faculty training system of the hospital, there is a lack of special training for improving lecturing skills.

Taking into account that lecturing skills are essential teaching skills for clinical teachers, they have a positive effect on achieving good teaching effects and teaching innovation. Therefore, the Teaching and Training Department set up a continuous project of lecturing skills improvement for clinical teacher to make up for their shortcomings. In addition, the improvement of lecturing skills can not be done at one go so the theoretical learning is just one step. The application of what had learned in clinical education and effect transference are the standard for evaluating teaching ability, so this project adopts the “intensive training + practice transference” model to study the effect of the project.

## Methods

**Fig. 1:**
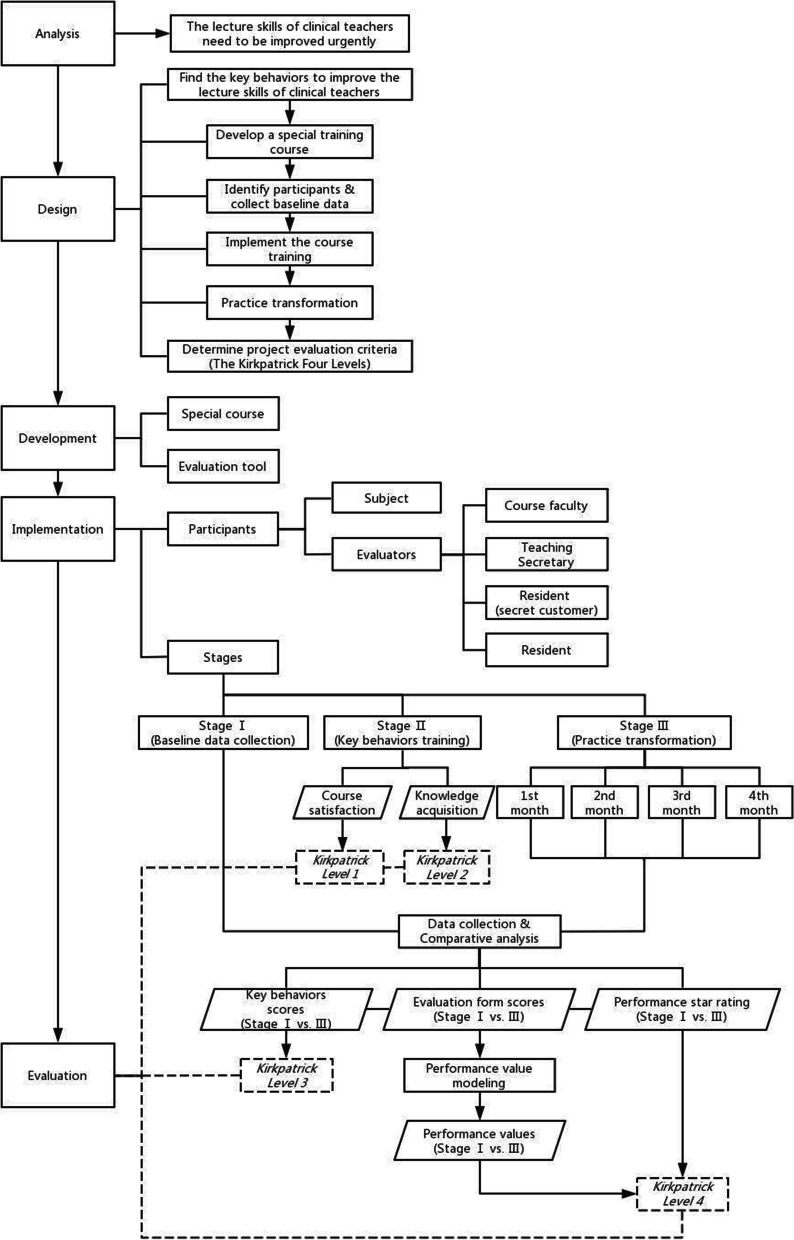
The road map of the continuous improvement project of lecturing skills for clinical teachers

This project is based on the classic instructional design ADDIE model, which is a method of systematically developing training projects. The ADDIE model has been used to help educators develop curriculum in many fields, including in medical education [5–9]. The five phases of the model are analysis, design, development, implementation, and evaluation. According to the ADDIE model, the project team conducts the research on the continuous improvement project of lecturing skills for clinical teachers in residency standardized training from five parts: requirements analysis, project design, development of the training course and evaluation tools, project implementation, and effect evaluation, as shown in Fig. 1. They are inseparable. Analysis and design are the precondition, development and implementation are the core. The evaluation is the guarantee for the success of the whole project, and it will run through the entire process. The project team uses the full-process evaluation to ensure the quality of the course training and practice transformation process.

### Analysis

The Teaching and Training Department set up the project team, including two staff of the department and 6 clinical teachers with strong teaching ability and rich teaching experience who are picked as the faculty team to jointly conduct project design and development. The faculty used the “Teaching Objective ABCD Approach” [10, 11] to conduct internal discussions and sample surveys on curriculum development requirements. The results are shown in Table 3.

**Table 3:** Survey results of the “Teaching Objective ABCD Approach”

Item	Questionnaire Description	Survey Results
A	Audience (Who is the subject?)	The clinical teachers in Shanghai East Hospital.
B	Behavior (What do you expect them to do?)	Master the theoretical knowledge of lecturing skills through training, and use them in teaching practice to improve the teaching level and the quality of small lectures.
C	Condition (Under what conditions does the behavior happen?)	The application of lecturing skills will be occurred in the small lectures for the residents.
D	Degree (To what extent does the behavior need to be completed? What standard is reached?)	To obtain the compliment from the teaching secretary and the residents of the department.

After discussion, the faculty believes that it is necessary to develop the special course of lecturing skills and implement training. The training object of CTPT course is the clinical teachers of Shanghai East Hospital. The learning target is to help them master the theoretical knowledge of lecturing skills through course training, and use the knowledge in teaching practice (such as small lectures) to improve their lecturing skills, and obtain the compliment from both the teaching management staff and the residents. The lecturing skills of clinical teachers are measured by the multi-dimensional scoring from the course faculty, the teaching secretary of the department, the residents attending the lectures and themselves. The performance values of their small lectures should be improved, and verified to be significant.

### Design

After clarifying the necessity and goals of the project based on the preliminary analysis, the faculty team began to design the project. They used Brain Storm Co-Create (BSCC) of expert group to study the key behaviors of small lecture teaching. The members of the expert group are composed of senior management staff of the hospital’s teaching department, winners of various lecture competitions over the years, clinical teachers with rich teaching experience and praised by students and residents’ representatives. Through BSCC, the key behaviors of clinical teachers with excellent lecturing skills in small lectures could be derived. Then the faculty team conducted open interviews with clinical teachers for these key behaviors and obtained a relatively unanimous agreement. Then five key behaviors (content, beginning, main body, ending and presentation) were determined to be the core elements throughout the whole project, that the follow-up course development, training development, and effect evaluation were based on.

According to the 5 key behaviors, the faculty developed a targeted teacher training course “Clinical Teacher Presentation Training” (CTPT), and recruited and determined the project participants and the evaluators. The subjects refer to the clinical teachers who give small lectures to residents, and the evaluators involve the faculty, the teaching secretary, the residents (secret customers) and other unwitting residents. In the Stage I, the evaluators collected the baseline data of lecturing ability of the subjects, then the subjects received the CTPT training and their satisfaction to CTPT and knowledge acquisition data were collected in the Stage II. After entering the Stage III of practice transference, the subjects carried out small lectures once a month for residents for 4 months, and the evaluators conducted continuous evaluations. Use the data obtained throughout the process to analyze the project effect. According to the Kirkpatrick Four Levels training evaluation model, the evaluations were carried out from the reaction level, the learning level, the behavior level and the result level [12–22], and the evaluation method is Triangulation [23]. The evaluation contents include the CTPT training satisfaction, knowledge acquisition, application of the 5 key behaviors at work, and improvement of lecture quality. Compare and analyze the data from multiple levels and multiple dimensions to verify the effect of the project.

**Fig. 2:**
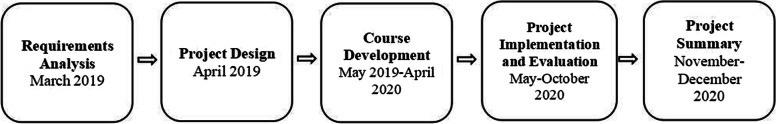
The time schedule of lecturing skills continuous improvement project for clinical teachers

The project execution time is from March 2019 to December 2020, as shown in Fig. 2.

### Development

#### Training course development

In May 2019, According to Gagne’s Principles of Instructional Design [24–27], the faculty developed the CTPT course. First, the five key behaviors (content, beginning, main body, ending and presentation) of small lecture were determined to be the main teaching content, and eight lessons were set up according to the content. Secondly, in order to facilitate the learning and mastering of knowledge points, the faculty summarized and summed up 14 tools to improve lecturing skills. Then, it was determined to give the lessons through the form of “workshops”, which could combine classroom lectures, interactive discussions, and practical operations. Besides, learning activities such as on-site investigations, group discussions, case analyses, and scenario simulations were designed in each lesson to help trainees apply these tools. Finally, a series of course supporting materials were developed, including course schedule (shown in Table 4), slides, provider manual, instructor manual, course feedback form, course certificate, course LOGO and poster, faculty badge, etc. Among them, the slides, the instructor manual and the provider manual have respectively obtained three national copyright certifications. The total duration of the course is 4 h and the instructor-to-student ratio is 1:10.

**Table 4:** CTPT course schedule

Time	LESSON
13:00–13:30 (30 min)	**LESSON 1 WARM UP** 1. Faculty Introduction 2. Trainee Self-introduction 3. Group Showtime (Video Recording)
	**LESSON 2 COURSE INTRODUCTION** 1. Course Introduction: Agenda\ Schedule\Content 2. Small Survey: What is a good lecture? 3. Curriculum Framework
13:30–13:45 (15 min)	**LESSON 3 KEY 1: CONTENT** 1. Tool 1: Content Range “My territory” 2. Tool 2: Teaching Object “Teach students in accordance with their aptitude” 3. Tool 3: Content Accuracy 4. Case Discussion & Case Demonstration 5. Review Tool 1–3
13:45–14:15 (30 min)	**LESSON 4 KEY 2: BEGINNING** 1. Tool 4: Put Question 2. Tool 5: Tell Story 3. Tool 6: Use Data 6. Case Discussion & Case Demonstration 4. Case Video 1 (5 min) 5. Group Exercise: Scenario Simulation
14:15–15:05 (50 min)	**LESSON 5 KEY 3: MAIN BODY** 1. Tool 7: Induction (3 types) 2. Tool 8: Imagination 3. Exercise: Lateral Thinking 4. Tool 9: Case Study (3 types) 5. Case Discussion & Case Demonstration 6. Group Exercise: Scenario Simulation
15:05–15:15 (10 min)	BREAK
15:15–15:30 (15 min)	**LESSON 6 KEY 4: ENDING** 1. Tool 10: Summarize with Numbers 2. Tool 11: 3F Model (Fact, Finding, Future) 3. Case Discussion & Case Demonstration 4. Group Exercise: Scenario Simulation 5. Review Tool 4–11
15:30–16:30 (60 min)	**LESSON 7 KEY 5: PRESENTATION** 1. “Group Showtime” Videos Review 2. Tool 12: PPT Production (3 principles) Case Video 2 (1 min) 3. Tool 13: Stage Performance (4 aspects) Body;Gesture;Eye Contact;Voice & Intonation Case Video 3 **(**4 min) 4. Tool 14: Socrates’ Questions and Answers Case Video 4 (5 min) 5. Case Discussion & Case Demonstration 6. Group Exercise: Scenario Simulation 7. Review Tool 12–14
16:30–17:00 (30 min)	**LESSON 8 CONCLUSION** 1. Case Video 5 (4 min) 2. The Core Quality of the Lecturers 3. Ending (Use Tool 10 & 11) 4. Course Feedback Survey

In the trial phase of the course (August 2019 to April 2020), the faculty conducted 10 trial lectures and completed 5 material revisions. They monitored the quality and continuously improved the curriculum development from the evaluation feedback of the developers, the instructors and the trainees. The course developers and instructors used the “Curriculum Development Evaluation Tool” (see Additional file 1) for self-evaluation; The trainees used the “Course Feedback Form” (see Additional file 2) to give their opinions and suggestions.

#### Evaluation tool development

In order to improve the efficiency of the evaluation, all scoring and star rating data collection in this project are completed on the mobile phone using online questionnaires or apps.

##### Star rating

During the course training of CTPT, the subjects’ knowledge acquisition of the 5 key behaviors were scored with stars by the faculty team. During the practice transference stage, the subjects were assigned to give small lectures to the residents once a month, and their overall performance was also evaluated with stars. The rating range is 0–5 stars.

##### Direct scoring

When collecting the baseline data of the subjects’ lecture skills and evaluating their practical application, the presentation of the 5 key behaviors in their small lectures was directly scored by the teaching secretaries. The scoring range is 0–100 points.

##### Score sheet

The 5 key behaviors are further refined, sub-items are set up with different weights, and the “Score Sheet of Small Lecture” is finally completed (Table 5). And then, through the correlation analysis and factor analysis of the questions of the score sheet, it is found that the questions are strongly correlated (see Additional file 3). And it indicates that the contents of these questions belong to the same category, and the score sheet is designed reasonably. And it was measured by Cronbach’s alpha as well. Cronbach’s alpha is 0.923, indicating that these 12 questions have high internal consistency. This score sheet is used in the case of collecting the baseline data of the subjects’ lecturing skills (Stage I) and evaluating the skills during practice transference (Stage III). The score is used as a data source for the subjects’ small lecture performance values modeling, and also represents the overall quality of their small lectures.

**Table 5:** Score sheet of small lecture for residency standardized training in Shanghai East Hospital

Item	Weights	Contents	Weights	Score (0–100)
Content	20	Full course preparation	5	
		Theme and content are suitable for residents’ characteristics and training requirements	5	
		Clear teaching goals, accurate content selection, and new progress	10	
Beginning	15	Theme is clear	5	
		Fascinating at the beginning	10	
Main body	20	The main content is substantial and contains useful knowledge	10	
		Skillful use of teaching skills	10	
Ending	15	High profile summary	10	
		Sublimation at the end, with profound meaning	5	
Presentation	30	PPT production	10	
		Proper etiquette	10	
		Full classroom interaction, focusing on critical clinical thinking	10	

### Implementation

#### Participants determination (May 2020)

##### Subjects

Ninety-nine clinical teachers from different departments were recruited through voluntary registration.

##### Evaluators

The project includes the following 4 types of evaluators.

*Course faculty*: 8 instructors also the developers of CTPT.

*Teaching secretaries*: In the teaching management structure of the residency standardized training program of Shanghai East Hospital, the teaching secretaries are the clinical physicians with rich teaching experience and enthusiasm designated by the department directors. In addition to completing the clinical work, they also need to assist in the management of teaching and training work of the department.

After the project recruitment, 22 teaching secretaries in the departments where the subjects are belonged are selected as peer evaluators. All of them have the following characteristics: Excellent teaching background (a medical bachelor degree or above, a professional title of attending physician for 5 years or above, and a qualification as lecturers at Tongji University School of Medicine); Good lecturing skills; Understand the project content and scoring rules; Familiar with the background and the teaching level of the subjects.

*Residents (secret customers)*: Refers to 30 residents randomly selected as mystery guests in related disciplines, and they participated in the effect evaluation anonymously.

*General residents*: Refers to the residents who are in rotation in the departments where the subjects belonged during the project, and they are unaware of the project.

#### Implementation stages

##### Stage I: baseline data collection (May 2020)

Before the start of data collection, the project team held a project briefing meeting. A unified task explanation and scoring standard training were conducted for the teaching secretaries and the residents (secret customers). The clarified tasks and guaranteed incentives enhanced the reliability and validity of the evaluation results. Subsequently, the subjects completed a small lecture in their departments, and the teaching secretaries and residents (secret customers) evaluated the quality of their small lectures, including scoring the 5 key behaviors, the Score Sheet of Small Lecture and the comprehensive performance star rating, then the teaching secretaries set the expected values.

##### Stage II: key behaviors training (June 2020)

The 99 subjects were divided into 6 sessions for the intensive training of CTPT, and about 16 subjects in each session were taught by 2 instructors in the classroom. The instructors taught in accordance with uniform teaching standards with the uniform teaching materials in the teaching process to ensure the standardization and homogeneity of the training. They also observed the subjects’ performance in the classroom and made a star rating according to their knowledge acquisition of the five key behaviors. After the training, the subjects filled out the course feedback form.

##### Stage III: practice transference (July–October 2020)

The design of this stage is to promote the behavior transference learned from the training in stage II to real clinical teaching. This stage is divided into 4 practice transference phases. Each phase is 1 month, and the practice transference is continuously carried out for 4 months. The subjects need to use the knowledge and skills to improve their lectures over a period of time, and gave a small lecture to the residents each month, lasting for 4 months. The teaching secretaries, the residents (secret customers) and general residents participated in the small lectures and completed their own assessment tasks, including direct scoring of the 5 key behaviors, Score Sheet of Small Lecture and comprehensive performance star ratings.

### Evaluation

The effect evaluation of this project is according to the Kirkpatrick Four Levels, which implements from the reaction level, the learning level, the behavior level and the result level. And the evaluation data of the five key behaviors were collected from the full-process (before, during, and after training, and practice transference stage) and multi-dimensional roles (course instructors, teaching secretaries, residents, and the subject themselves), as shown in Table 6.

**Table 6:** Kirkpatrick Four Levels evaluation of the project

Level	Content	Method
Level 1 Reaction	Training satisfaction of the CTPT course	After the CTPT course, the subjects fill out the course satisfaction questionnaire.
Level 2 Learning	Knowledge acquisition of the 5 key behaviors	a. During and after the CTPT course, the faculty give star ratings to the subjects; b. Before and after the CTPT course, the subjects directly score their own 5 key behaviors. Compare the self-efficacy [28–32] scores before and after by t-test.
Level 3 Behavior	Application of the 5 key behaviors in small lectures for residents	a. The teaching secretaries directly score the subjects’ 5 key behaviors in Stage I (Baseline data collection) and Stage III (4 phases of practice transference). Compared the data before and after by t-test. b. Before the CTPT course and after practice transference, the subjects directly score their own 5 key behaviors. Compare the self-efficacy scores before and after by t-test.
Level 4 Result	The improvement of the subjects’ small lectures	In Stage I (Baseline data collection) and Stage III(4 phases of practice transference): a. The teaching secretaries, the residents (secret customers) and general residents use the “Score Sheet of Small Lecture” to assess the subjects’ performance of small lectures, and compared the data before and after. b. Use the above data to model the performance value of the small lecture. Compare the performance values before and after by t-test. c. Carry out star rating for the subjects’ overall performance of small lectures. Compare the data before and after by t-test. d. Make correlation analysis on the change values of both performance values and star rating of the subjects’ overall performance by t-test.

The data analysis of this project adopts descriptive statistics, t-test, correlation analysis, analysis of variance, hypothesis testing

In this test, * as *P* < 0.05 indicating a statistical difference, ** as *P* < 0.01 and *** as *P* < 0.001 indicating a statistically significant difference

## Results

### General conditions of subjects

The general conditions of the 99 s are as follows: ①The gender ratio is relatively balanced; ②The age distribution is mainly concentrated in 30–49 years old, accounting for 87.88% (87/99), mainly young and middle-aged teachers; ③In terms of educational background, Master’s degree accounts for nearly half (49/99); ④The years of teaching experience are mainly concentrated in 1–9 years and 10–19 years, accounting for 42.42% (42/99) and 35.35% (35/99) respectively, and all of them have teaching experience. ⑤In terms of professional distribution, personnel in internal, surgical, gynecology, pediatrics, emergency and auxiliary departments are involved, and the distribution is relatively wide. See Table 7.

**Table 7:** Project subjects: general condition of the clinical teachers (*n* = 99)

Item	Sort	*n*(%)
Gender	Male	43(43.43)
	Female	56(56.57)
Age	20–29 years old	9(9.09)
	30–39 years old	62(62.63)
	40–49 years old	25(25.25)
	50–59 years old	3(3.03)
Educational background	Bachelor degree	25(25.25)
	Master’s degree	49(49.49)
	Doctoral degree	25(25.25)
The years of teaching experience	1–9 years	42(42.42)
	10–19 years	35(35.35)
	20–29 years	21(21.21)
	30-39 years	1(1.01)
Discipline	Internal Medicine	35(35.35)
	Surgical department	14(14.14)
	Obstetrics and Gynecology	7(7.07)
	Emergency Department	7(7.07)
	Stomatology Department	5(5.05)
	Anesthesiology Department	5(5.05)
	Rehabilitation Department	4(4.04)
	Radiology Department	6(6.06)
	Pediatric Department	5(5.05)
	Otorhinolaryngology Department	2(2.02)
	Medical Laboratory	5(5.05)
	ECG	4(4.04)

### Kirkpatrick four levels evaluation results

#### Level 1 reaction

##### Course satisfaction

The Course Feedback Form is used to investigate the subjects’ satisfaction with the CTPT course. The form adopts Likert-type scales, including five aspects: overall satisfaction, the faculty, course implementation, facilities and equipment, and net promoter score (NPS). The results show that the overall satisfaction of the course is 100% (99/99), and the NPS has reached 100% (99/99). It shows that the course has been highly accepted by all the subjects, see Table 8 for details.

**Table 8:** Satisfaction survey of the CTPT course (*n* = 99)

Item	Very Satisfaction/Strongly Agree	Satisfaction/Agree	General	Dissatisfaction/Disagree	Very Dissatisfaction/ Strongly Disagree
1. Overall satisfaction	75(75.76%)	24(24.24%)	0(0%)	0(0%)	0(0%)
2. The faculty:					
Full course preparation	81(81.82%)	18(18.18%)	0(0%)	0(0%)	0(0%)
The teaching method is easy to understand	77(77.78%)	22(22.22%)	0(0%)	0(0%)	0(0%)
Encourage students to participate in the course	80(80.81%)	18(18.18%)	1(1.01%)	0(0%)	0(0%)
Passionate	79(79.80%)	20(20.20%)	0(0%)	0(0%)	0(0%)
3. Course implementation:					
Course objectives are clearly stated	82(82.83%)	17(17.17%)	0(0%)	0(0%)	0(0%)
Meet my expectations for the course	75(75.76%)	24(24.24%)	0(0%)	0(0%)	0(0%)
Course exercises are well organized	76(76.77%)	23(23.23%)	0(0%)	0(0%)	0(0%)
Clear training materials	74(74.75%)	24(24.24%)	1(1.01%)	0(0%)	0(0%)
The course rhythm is appropriate	75(75.76%)	24(24.24%)	0(0%)	0(0%)	0(0%)
4. Facilities and equipment:					
The classroom environment is suitable for teaching	79(79.80%)	20(20.20%)	0(0%)	0(0%)	0(0%)
Smooth video equipment playback	69(69.70%)	25(25.25%)	5(5.05%)	0(0%)	0(0%)
5. I will recommend this course to others	64(64.65%)	35(35.35%)	0(0%)	0(0%)	0(0%)

#### Level 2 learning

##### Course instructor’s learning evaluation

**Fig. 3:**
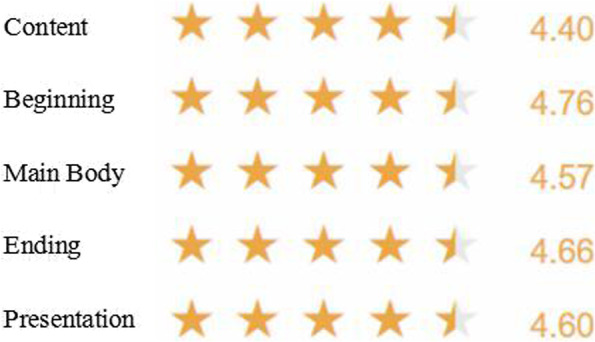
The star rating of the subjects’ knowledge acquisition of the 5 key behaviors scored by the faculty

During the course training of CTPT, the subjects acquired knowledge of the 5 key behaviors of lecture skills through observation, discussion, training and demonstration. The faculty gave a star rating (0–5 stars) according to the subjects’ performance in the classroom. The results showed that the understanding and acceptance of the 5 key behaviors of the 99 clinical teachers were all above 4 stars, indicating that the knowledge related to the key behaviors is well acquired (Fig. 3).

##### Subjects’ self-efficacy

In this level, the faculty compared the subjects’ self-efficacy scores before the training (May 2020) and after the training (June 2020), and the scores of 5 key behaviors were improved. After the difference test, the results showed statistical significance, *P* < 0.05. See Table 9.

**Table 9:** Test the subjects’ self-efficacy values before and after training($\overline{x}\pm s$)

Key Behaviors	Stage I (May 2020)	Stage II (June 2020)
Key Behavior 1: Content	80.71 ± 13.58	83.41 ± 8.11^*^
Key Behavior 2: Beginning	75.54 ± 15.42	83.97 ± 7.75^***^
Key Behavior 3: Main Body	78.35 ± 14.39	84.41 ± 7.66^***^
Key Behavior 4: Ending	76.33 ± 14.48	85.23 ± 7.55^***^
Key Behavior 5: Presentation	76.02 ± 14.85	84.26 ± 7.84^***^

Comparison of the self-efficacy values before and after training: ^*^ is *P*<0.05, ^***^ is *P*<0.001

The changes in self-efficacy after the training shows the training effect of the course. This CTPT course training has significantly improved the subjects’ 5 key behaviors of small lecture, which shows that the knowledge acquisition of the 5 key behaviors is also very good from the subjects’ self-evaluation.

#### Level 3 behavior

##### Teaching secretary scoring for 5 key behaviors

**Fig. 4:**
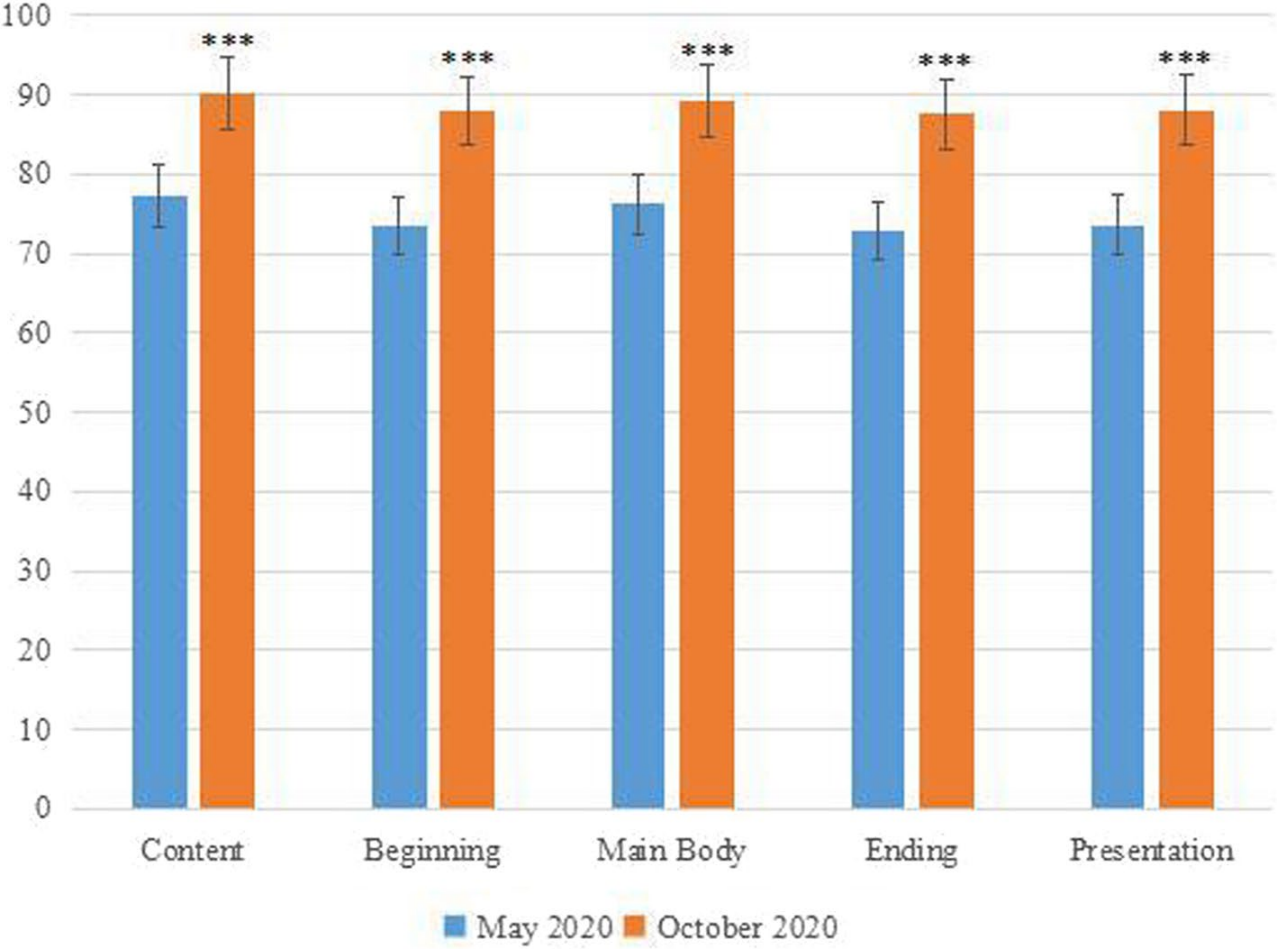
Comparison of the scores of the subjects’ 5 key behaviors before and after the project made by the teaching secretaries (*** is *P*<0.001)

In the real practice tasks, the teaching secretaries observed and scored the 5 key behaviors to the subjects’ performance during the small lectures. When comparing the secretaries’ marking on the 5 key behaviors of the subjects between the Stage I and Stage III, showing that all the five scores were improved, with an average increase of 14.12 points. At the same time, a difference test was carried out, and the result showed that the changes in the five scores were statistically significant, *P* < 0.001, as shown in Fig. 4. It shows that through this project, clinical teachers have used the 5 key behaviors in their small lectures and their skills are significantly improved and the 5 key behaviors have been successfully practiced and transferred to actual teaching work.

##### Subjects’ self-efficacy

In this level, the faculty compared the subjects’ self-efficacy scores before the training (May 2020) and after the practice transference stage (October 2020), and the subjects’ self-efficacy scores of 5 key behaviors were improved, with an average increase of 9.31 points (9.31%). After the difference test, the result showed statistical significance, *P*<0.001. See Table 10.

**Table 10:** Test the subjects’ self-efficacy values before training and after practice transference ($\overline{x}\pm s$)

Key Behaviors	Stage I (May 2020)	Stage III (October 2020)
Key Behavior 1: Content	80.71 ± 13.58	87.5 ± 7.01^***^
Key Behavior 2: Beginning	75.54 ± 15.42	86.15 ± 8.37^***^
Key Behavior 3: Main Body	78.35 ± 14.39	87.20 ± 7.72^***^
Key Behavior 4: Ending	76.33 ± 14.48	86.03 ± 8.15^***^
Key Behavior 5: Presentation	76.02 ± 14.85	86.63 ± 8.31^***^

Comparison of the self-efficacy values before training and after practice transference: ^***^ is *P*<0.001

Through the difference test of the subjects’ self-efficacy scores before and after the project, it is found that the subjects’ self-evaluation of the 5 key behaviors before and after the project is significantly different, indicating that from the perspective of the subjects, this project significantly improved their skill levels of the 5 key behaviors.

#### Level 4 results

##### Rating by three types of evaluators

**Fig. 5:**
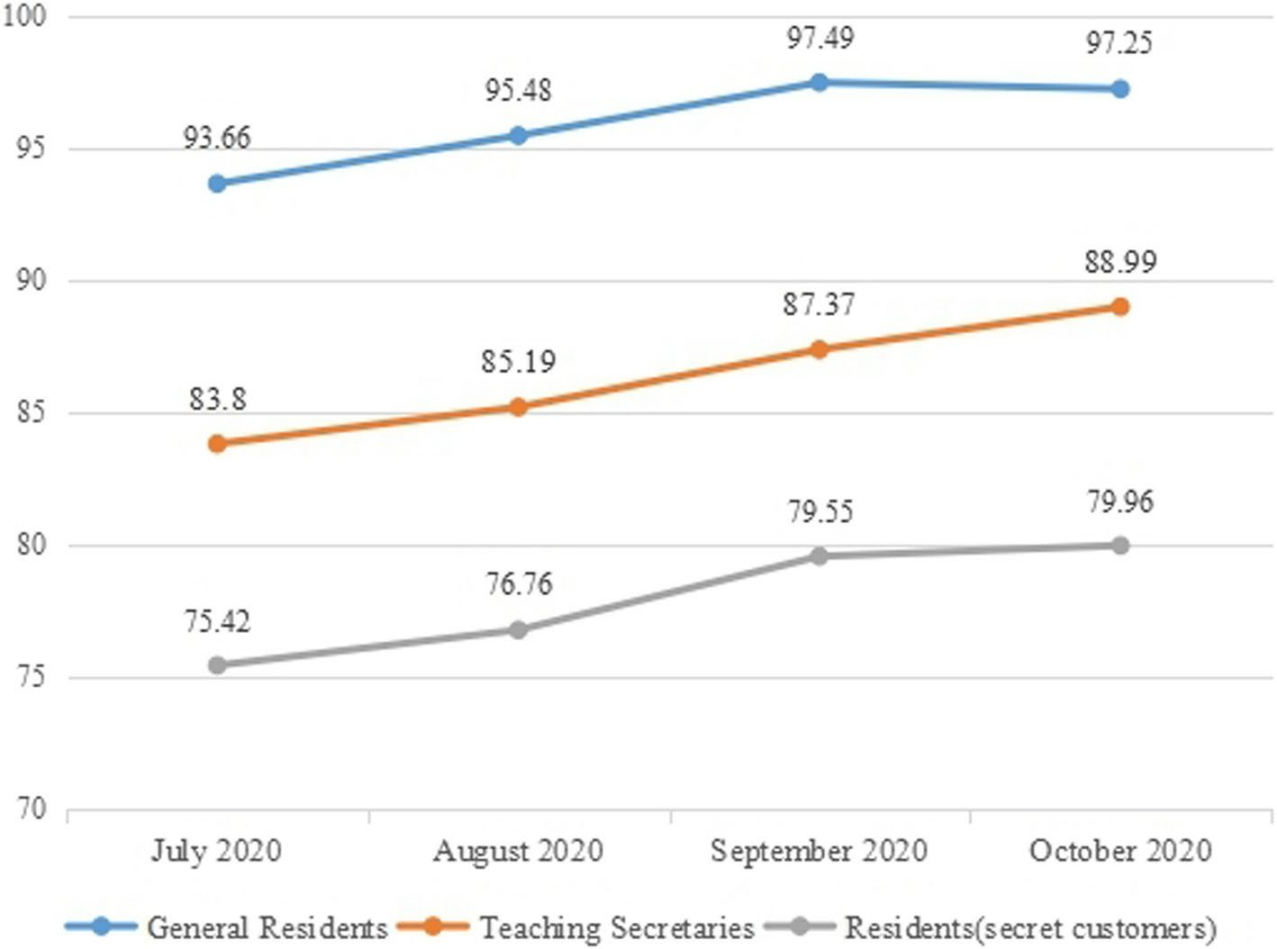
The subjects’ scores of the Small Lectures Score Sheet given by different evaluators at the practice transference stage

By analyzing the changes in the subjects’ scores of the Small Lecture Score Sheet at the practice transference stage by different types of evaluators, it is found that the score trends made by the three types of evaluators are the same, with an upward trend, as shown in Fig. 5. It shows that through training and working place practice, three types of evaluators all believed that the lecturing skills of the subjects had been improved.

A further comparison of the scores given by the three types of evaluators reveals that the scores given by the general residents are much higher than those given by the teaching secretaries and the residents (secret customers). The reasons may be: ① The general residents have not been trained of scoring standards of the project; ②The general residents don’t know about the project and the purpose of their scores quite well, and there is also a situation of favor points.

There are also differences in the scores of the teaching secretaries and the residents (secret customers). The reason for the difference lies in the fact that there are objective differences in identities and teaching experience between the teaching secretaries and the residents (secret customers), which leads to differences in cognition and understanding of the subjects and lecturing skills. Therefore, even if they have been explained for the score sheet, they still have differences in scoring, which is within a reasonable range.

Based on the above situation, in order to ensure the reliability and validity of the project evaluation, the project team conducted performance value modeling. After removing the scores given by the general residents, reassigned the weights of the scores given by the teaching secretaries and the residents (secret customers). After investigation and discussion, the project team believes that the evaluation of the subjects’ small lectures should be much more inclined to the opinions of the residents (secret customers), so the final performance value model is determined:

$$\mathbf{Performance}\ \mathbf{Value}=\mathbf{55}\%\mathbf{the}\ \mathbf{score}\ \mathbf{given}\ \mathbf{by}\ \mathbf{the}\ \mathbf{resident}\ \left(\mathbf{secret}\ \mathbf{customer}\right)+\mathbf{45}\%\mathbf{the}\ \mathbf{score}\ \mathbf{given}\ \mathbf{by}\ \mathbf{the}\ \mathbf{teaching}\ \mathbf{secretary}.$$

##### Significant improvement of performance value

**Fig. 6:**
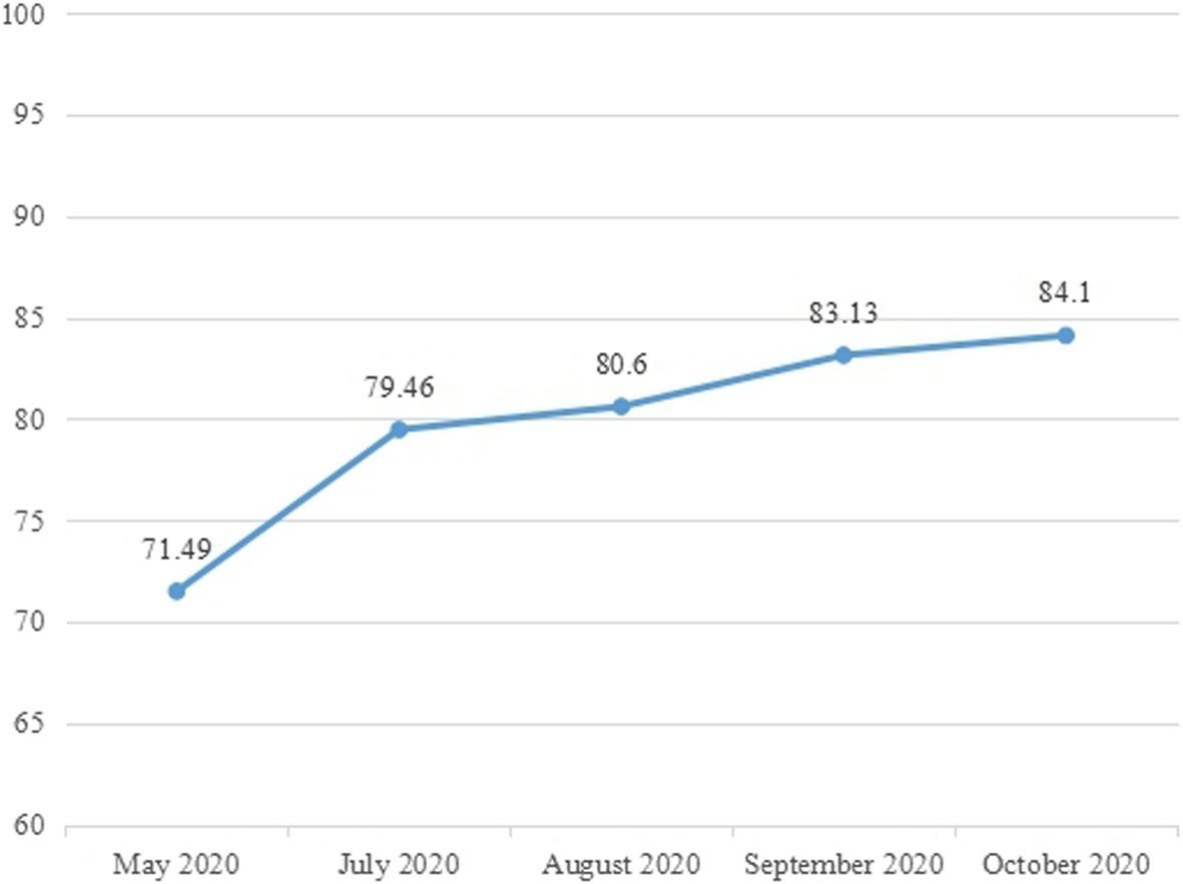
The subjects’ performance values of small lectures in different stages

Analyzing the changes in the performance value data, it can be found that from the pre-training to real clinical teaching, the performance value continues to increase, from 71.49 points to 84.10 points, and totally increased 12.61 points. Among the changes in the performance value at each stage, the largest change is from May (before training) to July (the first month of practice transference), with a change of 7.97, while in the next 3 months, the change is relatively small (Fig. 6).

At the same time, the performance value was tested for difference, and the result was statistically significant, *P* < 0.001 (Table 11). It shows that the implementation effect of this project is significant and the lecturing skills and lecture quality have been effectively improved through this project.

**Table 11:** Test the subjects’ performance values of small lectures before and after training ($\overline{x}\pm s$)

Item	Stage I (May 2020)	Stage III (October 2020)
Performance values	71.49 ± 9.21	84.10 ± 7.51^***^

Comparison of the performance values before and after training: *** is *P*<0.001

##### Continuous improvement of star rating of the subjects’ overall performance

**Fig. 7:**
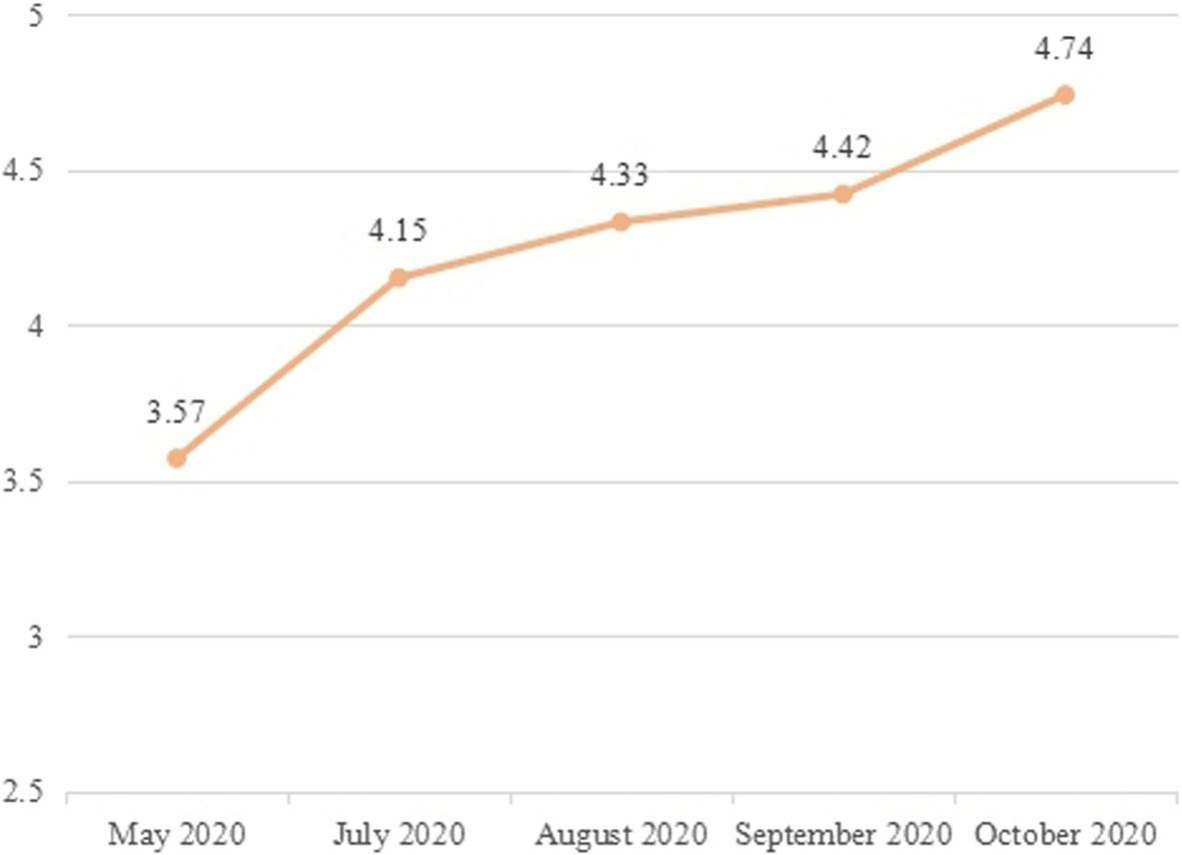
The star rating of the subjects’ small lectures in different stages made by the teaching secretaries

Analyzing the star rating score of the subjects’ overall performance in their small lectures which is given by the teaching secretaries, it can be found that from the pre-training to the practice transference stage, the star rating score continued to increase, with a total increase of 1.17 points. Among the data changes at each stage, the largest change was from May (before training) to July (the first month of practice transference), with a change of 0.58. While in the last 3 months, the changes were small, as shown in the Fig. 7.

At the same time, a difference test was performed, and the test results were statistically significant, *P* < 0.001, as shown in Table 12 It shows that the overall performance of the subjects’ small lectures has been significantly improved through this project.

**Table 12:** Test the subjects’ star rating made by the teaching secretaries before and after training ($\overline{x}\pm s$)

Item	Stage I (May 2020)	Stage III (October 2020)
Star rating of different stage	3.57 ± 0.46	4.74 ± 0.44^***^

Comparing the data before and after training: ***is *P*<0.001

##### Correlation analysis of the performance value and the star rating

Comparing the changes of the performance value and the star rating for the subjects’ overall performance scored by the teaching secretaries, we found that:

- ① The two trends are consistent and continue to improve, which indicates that the subjects’ lecturing skills and overall performance of small lectures have been effectively improved after the training and practice transference of the project.
- ② Testing and analyzing the difference in performance values at different stages, it is found that there is a significant difference between the improvement in the “July–May” stage and the “August–July” stage; there is a significant difference between the improvement in the “August–July” stage and the “September–August” stage; there is a significant difference between the improvement in the “October- September “stage and the “September–August” stage. Among them, the “July–May” stage has the largest increase, as shown in Table 10.
- ③ Testing and analyzing the difference in star rating at different stages, it is found that there is a significant difference between the improvement in the “July–May” stage and the “August–July” stage; there is no significant difference between the improvement in the “August–July” stage and the “September–August” stage. There is a significant difference between the improvement in the “October–September” stage and the “September–August” stage. Among them, the “July–May” stage also has the largest increase, as shown in Table 13.

**Table 13:** Test of the performance value and the star rating given by the teaching secretaries before training and after practice transference ($\overline{x}\pm s$)

Item	July–May	August–July	September–August	October–September
The difference between the performance values of the stages	7.75 ± 6.81	1.51 ± 3.24***	2.58 ± 3.45*	1.08 ± 4.49**
The difference between the star ratings of the stages	0.57 ± 0.62	0.19 ± 0.53***	0.09 ± 0.46	0.33 ± 0.49**

Compare with the former stage:* is *P*<0.05, ** is *P*<0.01, *** is *P*<0.001

To sum up, the most significant improvement both in the performance value and the star rating at different stages is the “July–May” stage, which indicates that the greatest improvement occurred in the period between the time before the training to the first month of practice transference.

## Discussion

1. The lecturing skills of clinical teachers can be effectively improved through training. The data proves that the CTPT course developed by the faculty of Shanghai East Hospital has a positive effect on improving the lecturing skills of clinical teachers, and it could be promoted as a special course for teaching ability in the faculty developing training in the hospital.
  Among the subjects of the project, although the lecturing skills of clinical teachers with different teaching experience have been improved, those with less than 20 years of teaching experience accounted for 77%. Therefore, whether this course is more helpful for the clinical teachers with less teaching experience or not is unverified, and further research is needed. In the next step, more detailed stratified researches could be done on the background factors of clinical teachers, and the instructional design of the courses could be optimized according to the different backgrounds of clinical teachers, and then the efficiency of training could be further improved.
2. The research has been proved to be effective from the Kirkpatrick Four Levels. In this project, from the course training to practice transference, the performance values of the subjects at each stage are continuously improved. So the course training is not the end of skill improvement, and we should pay more attention to the application and conversion of the knowledge to the actual teaching scene. The project design of “course training + practice transference” is a better choice.
  This project includes a 4-phases practice transference stage that lasts totally 4 months. The data shows that the performance values changes in each phase are not the same (the greatest improvement occurred in the period between the time before the training to the first phase of practice transference), which may mean the evaluation process needs to be refined and continued over a longer period to determine the most appropriate practice transference period.
3. There is reference significance for the faculty development and training research projects in the Residency Standardized Training in the future to incorporating peer evaluation [33–36] (such as teaching secretaries) into teaching practices.
  Considering that the presence of a teaching secretary may cause clinical teachers to pay more attention to their teaching and preparation for the lectures, Shanghai East Hospital has begun to try to incorporate teaching secretaries as observers into regular teaching activities, and encouraged them to carry out peer evaluation.
  Next, it is necessary to further explore various influencing factors that promote performance conversion, including course training, the settings of practice transference stages and teaching interventions, and study their effects.
4. This project studies the improvement of lecturing skills of clinical teachers in the hospital. As for the impacts and outcomes of the course on resident learning and standardized training, it is unclear yet, and it is worthy of further exploration in future research.

## Conclusion

Through the preliminary investigation, it is found that there are problems of poor quality of small lectures and lack of lecturing skills of the clinical teachers in the residency standardized training of Shanghai East Hospital. Therefore, the Teaching and Training Department has set up a project team to conduct research on the continuous improvement project of lecture. Based on the ADDIE model of instructional design, the team conducted the project in accordance with a process of analysis, design, development, implementation and evaluation. And the project team finally developed a targeted teacher training course “Clinical Teacher Presentation Training” (CTPT), based on the 5 key behaviors, “content”, “beginning”, “main body”, “ending” and “presentation”. After the 99 subjects received the intensive training and the 4-months’ real clinical teaching practice tasks, the project team conducted a multi-dimensional and full-process evaluation of the project from the reaction level, the learning level, the behavior level and the result level, according to the Kirkpatrick Four Levels training evaluation model. The results show that the training satisfaction of CTPT is 100%, the subjects have a high degree of knowledge acquisition, and the 5 key behaviors have been practically used in their small lectures. Comparing the data before training and after practice transference, it is found that the subjects’ 5 key behaviors scores made by teaching secretaries increased by an average of 14.12 points (14.12%), and the subjects’ self-efficacy scores increased by an average of 9.31 points (9.31%). The subjects’ small lecture performance values increased by an average of 12.61 points (12.61%). The star ratings of the overall performance have been improved by an average of 1.17 points (23.4%). All above the results are statistically significant (*P*<0.001). It is proved that the project effectively promoted the improvement of the clinical teachers’ lecturing skills and the quality of small lectures. The project goals have been achieved.

It is just a beginning of the research on the CTPT training course and the continuous improvement project. The Teaching and Training Department needs to carry out more teaching researches and explorations, and then the results can be applied to the improvement of the curriculum and the teacher training system, so that a closed-loop teaching management model of analysis, design, development, implementation and evaluation can be formed, which will help clinical teachers to improve their teaching skills more effectively.

## Supplementary Information

**Additional file 1.**

**Additional file 2.**

**Additional file 3.**

## Data Availability

The datasets used and/or analyzed during the current study are available from the corresponding author on reasonable request.

## Abbreviation

CTPT: Clinical Teacher Presentation Training

## References

[1] Wang C, Qi X, Chen X (2015). The establishment of China standardized residency training system. Zhonghua Yi Xue Za Zhi.

[2] Liu CX, Ouyang WW, Wang XW (2020). Comparing hybrid problem-based and lecture learning (PBL + LBL) with LBL pedagogy on clinical curriculum learning for medical students in China: a meta-analysis of randomized controlled trials. Medicine (Baltimore).

[3] Zeng HL, Chen DX, Li Q (2020). Effects of seminar teaching method versus lecture-based learning in medical education: a meta-analysis of randomized controlled trials. Med Teach.

[4] Ding C, Li S, Chen B (2019). Effectiveness of flipped classroom combined with team-, case-, lecture- and evidence-based learning on ophthalmology teaching for eight-year program students. BMC Med Educ.

[5] Tobase L, Peres HHC, Almeida DM (2018). Instructional design in the development of an online course on basic life support. Rev Esc Enferm USP.

[6] Fernandes RAML, Lima JTO, Silva BH (2020). Development, implementation and evaluation of a management specialization course in oncology using blended learning. BMC Med Educ.

[7] Kim S, Choi S, Seo M (2020). Designing a clinical ethics education program for nurses based on the ADDIE model. Res Theory Nurs Pract.

[8] Noh J (2020). Development and evaluation of a multimodality simulation disaster education and training program for hospital nurses. Int J Nurs Pract.

[9] Ab Latif R, Mat Nor MZ (2020). Using the ADDIE model to develop a Rusnani concept mapping guideline for nursing students. Malays J Med Sci.

[10] Xu X. Design of teaching objectives based on ABCD approach: a case study. Jiangsu Educ Res. 2013;(6):44–7. 10.13696/j.cnki.jer1673-9094.2013.06.010.

[11] Liu Z. ABCD of teaching objective description. Sichuan Educ. 2016;(4):33. 10.3969/j.issn.1005-1910.2016.04.021.

[12] Johnston S, Coyer FM, Nash R (2018). Kirkpatrick’s evaluation of simulation and debriefing in health care education: a systematic review. J Nurs Educ.

[13] Chellaiyan VG, Suliankatchi RA (2019). Health research methodology workshop: evaluation with the Kirkpatrick model. Natl Med J India.

[14] Dorri S, Akbari M, Dorri Sedeh M (2016). Kirkpatrick evaluation model for in-service training on cardiopulmonary resuscitation. Iran J Nurs Midwifery Res.

[15] Baines R, de Bere SR, Stevens S (2018). The impact of patient feedback on the medical performance of qualified doctors: a systematic review. BMC Med Educ.

[16] Abdulghani HM, Shaik SA, Khamis N (2014). Research methodology workshops evaluation using the Kirkpatrick’s model: translating theory into practice. Med Teach.

[17] Ostapchuk M, Patel PD, Miller KH (2010). Improving residents’ teaching skills: a program evaluation of residents as teachers course. Med Teach.

[18] Ragsdale JW, Berry A, Gibson JW (2020). Evaluating the effectiveness of undergraduate clinical education programs. Med Educ Online.

[19] Kim DH, Yoon HB, Sung M (2015). Evaluation of an international faculty development program for developing countries in Asia: the Seoul intensive course for medical educators. BMC Med Educ.

[20] Frye AW, Hemmer PA (2012). Program evaluation models and related theories: AMEE guide no. 67. Med Teach.

[21] LoVerde JA, Kerber C, Kisch T (2021). Comparison of lecture and manipulative teaching methods on learning and application to practice. Nurs Forum.

[22] Lee H, Song Y (2021). Kirkpatrick model evaluation of accelerated second-degree nursing programs: a scoping review. J Nurs Educ.

[23] Carter N, Bryant-Lukosius D, DiCenso A (2014). The use of triangulation in qualitative research. Oncol Nurs Forum.

[24] Buscombe C (2013). Using Gagne’s theory to teach procedural skills. Clin Teach.

[25] Gogineni H, Aranda JP, Garavalia LS (2019). Designing professional program instruction to align with students’ cognitive processing. Curr Pharm Teach Learn.

[26] Berger-Estilita J, Greif R (2020). Using Gagne’s “instructional design” to teach clinically applicable knowledge in small groups. Trends Anaesth Crit.

[27] Lo WL, Hsieh MC (2019). Teaching communication skills: using Gagne’s model as an illustration. Ci Ji Yi Xue Za Zhi.

[28] Bourne MJ, Smeltzer SC, Kelly MM (2021). Clinical teacher self-efficacy: a concept analysis. Nurse Educ Pract.

[29] Mendhi MM, Premji S, Cartmell KB (2020). Self-efficacy measurement instrument for neonatal resuscitation training: an integrative review. Nurse Educ Pract.

[30] Mohammadi F, Kohan S, Farzi S (2021). The effect of pregnancy training classes based on bandura self-efficacy theory on postpartum depression and anxiety and type of delivery. J Educ Health Promot.

[31] Huang XX, Mayer RE (2019). Adding self-efficacy features to an online statistics lesson. J Educ Comput Res.

[32] Li AT, Su YW (2014). Exploring the relationship between personality features and teaching self-efficacy in clinical nursing preceptors. J Nurs Res.

[33] Perera J, Perera J, Abdullah J (2009). Training simulated patients: evaluation of a training approach using self-assessment and peer/tutor feedback to improve performance. BMC Med Educ.

[34] Salih ARA (2013). Peer evaluation of teaching or ‘fear’ evaluation: in search of compatibility. High Educ Stud.

[35] Rees EL, Davies B, Eastwood M (2015). Developing students’ teaching through peer observation and feedback. Perspect Med Educ.

[36] García SAV, Stacy-Bates KK, Alger J (2017). Peer evaluation of teaching in an online information literacy course. Portal-Libr Acad.

