# Supplementary material for: A case study: a continuous improvement project of lecturing skills for clinical teachers in Chinese residency standardized training
Source: BMC Med Educ. 2022 Apr 11;22:265. doi: 10.1186/s12909-022-03311-z (PMC8996608; doi:10.1186/s12909-022-03311-z)
Supplement: Supplementary file 1 — Additional file 1. [file 12909_2022_3311_MOESM1_ESM.docx]

| **Curriculum Development Evaluation Tool** | | | | | | |
| --- | --- | --- | --- | --- | --- | --- |
| Course Structure | Need to be improved | | | | Excellent | Your Suggestion |
| Logical Order | 1 | 2 | 3 | 4 | 5 |  |
| Clarify the benefits that students can get after completing the course | 1 | 2 | 3 | 4 | 5 |  |
| Contact the students' past learning content and experience | 1 | 2 | 3 | 4 | 5 |  |
| There is a good transition between the various learning stages | 1 | 2 | 3 | 4 | 5 |  |
| Practice design is closely related to the actual situation | 1 | 2 | 3 | 4 | 5 |  |
| Variety of teaching methods and multimedia use | 1 | 2 | 3 | 4 | 5 |  |
| Course review/summary | 1 | 2 | 3 | 4 | 5 |  |
| Taking into account how the course content is transferred to work | 1 | 2 | 3 | 4 | 5 |  |
| Course Organization | Need to be improved | | | | Excellent | Your Suggestion |
| Appropriate abundance of materials | 1 | 2 | 3 | 4 | 5 |  |
| Course teaching is closely related to work tasks/procedures | 1 | 2 | 3 | 4 | 5 |  |
| The setting and expression of learning objectives are clear | 1 | 2 | 3 | 4 | 5 |  |
| Clearly defined related terms | 1 | 2 | 3 | 4 | 5 |  |
| There are clear instructions for the course content and materials | 1 | 2 | 3 | 4 | 5 |  |
| Special emphasis on key content | 1 | 2 | 3 | 4 | 5 |  |
| Time management | 1 | 2 | 3 | 4 | 5 |  |
| Course Materials | Need to be improved | | | | Excellent | Your Suggestion |
| Readability / legibility of the material | 1 | 2 | 3 | 4 | 5 |  |
| Use of work aids | 1 | 2 | 3 | 4 | 5 |  |
| Page numbers, spelling and grammar | 1 | 2 | 3 | 4 | 5 |  |
| How should the course be further improved?  Course Structure：  Course Organization：  Course Materials： | | | | | | |
