# Supplementary material for: A case study: a continuous improvement project of lecturing skills for clinical teachers in Chinese residency standardized training
Source: BMC Med Educ. 2022 Apr 11;22:265. doi: 10.1186/s12909-022-03311-z (PMC8996608; doi:10.1186/s12909-022-03311-z)
Supplement: Supplementary file 2 — Additional file 2. [file 12909_2022_3311_MOESM2_ESM.docx]

| **Clinical Teacher Presentation Training Course Feedback Form**  Thank you for taking the time to participate in this course training, and hope this course can help you effectively improve your lecturing skills. Please take the time to give feedback on the questionnaire and encourage us to continue to improve in the future. Thank you for your cooperation. | | | | | |
| --- | --- | --- | --- | --- | --- |
| **Item** | **Very Satisfaction/**  **Strongly Agree** | **Satisfaction/Agree** | **General** | **Dissatisfaction/**  **Disagree** | **Very Dissatisfaction/ Strongly Disagree** |
| 1.Overall satisfaction |  |  |  |  |  |
| 2.The faculty | | | | | |
| Full course preparation |  |  |  |  |  |
| The teaching method is easy to understand |  |  |  |  |  |
| Encourage students to participate in the course |  |  |  |  |  |
| Passionate |  |  |  |  |  |
| 3.Course implementation | | | | | |
| Course objectives are clearly stated |  |  |  |  |  |
| Meet my expectations for the course |  |  |  |  |  |
| Course exercises are well organized |  |  |  |  |  |
| Clear training materials |  |  |  |  |  |
| The course rhythm is appropriate |  |  |  |  |  |
| 4.Facilities and equipment | | | | | |
| The classroom environment is suitable for teaching |  |  |  |  |  |
| Smooth video equipment playback |  |  |  |  |  |
| 5.I will recommend this course to others |  |  |  |  |  |
| 6.Any comments and suggestions for this course? What are the shortcomings of this course? What needs to be improved? | | | | | |
