# Supplementary material for: A case study: a continuous improvement project of lecturing skills for clinical teachers in Chinese residency standardized training
Source: BMC Med Educ. 2022 Apr 11;22:265. doi: 10.1186/s12909-022-03311-z (PMC8996608; doi:10.1186/s12909-022-03311-z)
Supplement: Supplementary file 3 — Additional file 3. [file 12909_2022_3311_MOESM3_ESM.docx]

**Evaluation Scale Test**

Through the correlation analysis and factor analysis of the questions of the score sheet, it is found that the questions are strongly correlated. And it indicates that the contents of these questions belong to the same category, and the score sheet is designed reasonably, as shown in Table 1-1 and Table 1-2.

And it was measured by Cronbach's alpha as well. Cronbach's alpha is 0.923, indicating that these 12 questions have high internal consistency.

**Table 1-1.** Score sheet of small lecture correlation coefficient matrix

|  | Question 1 | Question 2 | Question3 | Question4 | Question5 | Question6 | Question7 | Question8 | Question9 | Question10 | Question11 | Question  12 |
| --- | --- | --- | --- | --- | --- | --- | --- | --- | --- | --- | --- | --- |
| Question1 | — |  |  |  |  |  |  |  |  |  |  |  |
| Question2 | 0.763^**^ | — |  |  |  |  |  |  |  |  |  |  |
| Question3 | 0.787^**^ | 0.752^**^ | — |  |  |  |  |  |  |  |  |  |
| Question4 | 0.819^**^ | 0.750^**^ | 0.761^**^ | — |  |  |  |  |  |  |  |  |
| Question5 | 0.677^**^ | 0.585^**^ | 0.705^**^ | 0.712^**^ | — |  |  |  |  |  |  |  |
| Question6 | 0.863^**^ | 0.777^**^ | 0.840^**^ | 0.791^**^ | 0.677^**^ | — |  |  |  |  |  |  |
| Question7 | 0.738^**^ | 0.566^**^ | 0.664^**^ | 0.719^**^ | 0.735^**^ | 0.772^**^ | — |  |  |  |  |  |
| Question8 | 0.833^**^ | 0.686^**^ | 0.785^**^ | 0.758^**^ | 0.734^**^ | 0.836^**^ | 0.801^**^ | — |  |  |  |  |
| Question9 | 0.629^**^ | 0.565^**^ | 0.679^**^ | 0.674^**^ | 0.666^**^ | 0.647^**^ | 0.695^**^ | 0.709^**^ | — |  |  |  |
| Question10 | 0.796^**^ | 0.678^**^ | 0.725^**^ | 0.753^**^ | 0.681^**^ | 0.767^**^ | 0.738^**^ | 0.834^**^ | 0.702^**^ | — |  |  |
| Question11 | 0.725^**^ | 0.598^**^ | 0.631^**^ | 0.630^**^ | 0.627^**^ | 0.680^**^ | 0.660^**^ | 0.751^**^ | 0.568^**^ | 0.717^**^ | — |  |
| Question12 | 0.787^**^ | 0.764^**^ | 0.740^**^ | 0.835^**^ | 0.745^**^ | 0.795^**^ | 0.755^**^ | 0.817^**^ | 0.701^**^ | 0.810^**^ | 0.732^**^ | — |

**Table 1-2.** Score sheet of small lecture factor analysis results

| Question | Factor Loading |
| --- | --- |
| Question1 | 0.907^**^ |
| Question2 | 0.815^**^ |
| Question3 | 0.877^**^ |
| Question4 | 0.759^**^ |
| Question5 | 0.861^**^ |
| Question6 | 0.793^**^ |
| Question7 | 0.912^**^ |
| Question8 | 0.843^**^ |
| Question9 | 0.913^**^ |
| Question10 | 0.874^**^ |
| Question11 | 0.777^**^ |
| Question12 | 0.902^**^ |
